# Innovative CF/PVC Foam Applicated for Automotive Synthetic Leather with High-Performance and Reduced VOC Emissions

**DOI:** 10.3390/ma17051076

**Published:** 2024-02-26

**Authors:** Hongfu Li, Ying Wu, Lingyan Wu, Changwei Cui, Kangmin Niu

**Affiliations:** School of Materials Science and Engineering, University of Science and Technology Beijing, Beijing 100083, China; wuying@ustb.edu.cn (Y.W.);

**Keywords:** PVC foam, automotive synthetic leather, carbon fiber, mechanical properties, VOC emission

## Abstract

Polyvinyl chloride (PVC) foam, valued for its mechanical and thermal properties along with cost-effectiveness, is extensively utilized across diverse industries. However, its high volatile organic compound (VOC) emissions hinder its adoption in eco-friendly synthetic leather. This study proposes a solution by optimizing the formulation design and foaming processes and achieving mechanical property enhancement via carbon-fiber-reinforced PVC composite foam (CF/PVC). The aim is to reduce PVC usage via enhancing its intrinsic properties. Systematic investigations were carried out on the impact of foaming raw materials, foaming processes, fiber content, and fiber length on the foaming performance, mechanical properties, and VOC emissions. The material formulation and process parameters were successfully optimized. Further assessment of various indicators such as the density, mechanical properties, and tear resistance of synthetic leather samples confirmed that the innovative CF/PVC foam developed in this study meets the requirements for automotive interior applications. Notably, the tensile strength and tear resistance of CF/PVC composite synthetic leather increased by 50% and 29%, respectively, compared to pure PVC, while VOC emissions decreased by 28%. It is anticipated that a more pronounced reduction in VOC emissions will be achieved in practical automotive interior leather applications when further considering the reinforcing effect of fibers, which leads to a reduction in PVC usage. The findings present a technical reference for innovative applications, aiming to enhance PVC foam performance and minimize emissions.

## 1. Introduction

Thermoplastic polyvinyl chloride (PVC) foam exhibits favorable mechanical properties, thermal insulation, wear resistance, chemical durability, and cost-effectiveness [1,2], contributing to its extensive applications in diverse sectors, including automotive interiors, fashion, home furnishings, etc. [3,4]. Particularly, in the domain of synthetic leather, the characteristics of rich color options, customizable patterns, and animal-friendly make PVC foam a preferred choice and an indispensable material in our daily life [5,6,7,8,9]. However, with the advancement of socioeconomic development and increased consumer awareness of health, as well as a growing emphasis on energy efficiency and environmental sustainability, the inherent high potential volatile organic compound (VOC) emissions associated with PVC, stemming from its chlorine-containing chemical structure and high content of additives, limit its potential applications in environments like automotive interiors, where VOC emissions could directly affect human health. For example, vinyl chloride (C_2_H_3_Cl) is used in the synthesis of PVC. The liver is mainly affected by exposure to air containing vapors of vinyl chloride, resulting in liver damage or impaired liver function [10]. Furthermore, it is highly likely to be carcinogenic for the people residing close to factories that produce vinyl chloride or within heavy VOCs [10,11,12,13]. Based on the evaluation of Wang’s work [14], it is recommended that PVC products should be stored in a well-ventilated place for at least 28 days before indoor use. Therefore, in the context of meeting basic performance requirements, reducing PVC usage while simultaneously lowering inherent VOC emission levels holds practical significance for green and health-oriented applications of PVC.

Addressing this challenge, optimizing material formulation and foaming processes is the most direct and efficient means to reduce PVC foam VOC emissions [15]. Furthermore, borrowing from the concept of fiber-reinforced composites in the aerospace industry, where high material-specific strength design can effectively achieve structural weight reduction, achieving high mechanical performance in PVC foam represents a potential and feasible approach to directly reducing PVC usage. Specifically, regarding material formulation and foaming processes, optimization can be achieved through the use of plasticizers, heat stabilizers, and related process parameters. For instance, Demir et al. [16] studied the effects of Ca/Zn stearate and organotin heat stabilizers and zeolite, CaCO_3_, cellulose, and luffa flours fillers and their concentrations on PVC foam morphology, foam density, compressive mechanical properties, and water uptake capacities. The morphology of the sample without any filler showed that the employment of Ca stearate and Zn stearate heat stabilizers instead of organotin stabilizers increases foam formation and decreases pore sizes and regularity in pore size distribution. Foams with organotin stabilizer were more resistant to heat than the ones with Ca/Zn stearate for long heating periods. Foams, including organotin-based heat stabilizers, have a compact structure. Samples containing zeolite, CaCO_3_, cellulose, or luffa flour had lower pore volume but higher Young’s modulus and stress values compared to unfilled samples. Shi et al. [17] investigated the effect of different plasticizers on PVC properties. The thermal stability, migration resistance, and transparency of PVC with tributyl citrate (TBC) were better than PVC with dioctyl phthalate (DOP) due to the good compatibility between TBC and Zn(Arg)_2_. The best properties were further achieved when a mixture of dioctyl terephthalate (DOTP) and TBC in a 1:1 ratio was used as a plasticizer for Zn(Arg)_2_-stabilized PVC. Additionally, the longer the carbon chain in citric acid esters, the better the thermal stability and transparency of PVC sample due to the hydroxyl group in citric acid esters. Santos et al. [18] introduced paraffin phase change materials to improve the thermal regulation performance of PVC foam synthetic leather. This addition increased the response time of synthetic leather to changes in environmental temperature, achieving a warming effect in winter and a cooling effect in summer, thereby enhancing comfort. It can be inferred that by selecting appropriate formulations of PVC additives and optimizing process parameters it is possible to achieve low VOC emissions by enhancing the stability of PVC.

For the modification of PVC to achieve high mechanical performance, the application of fiber-reinforced polymers and foaming materials to achieve high specific strength and realize PVC usage reduction is a burgeoning trend and research focus in the field [19,20]. Fiber-reinforced PVC foams can significantly enhance the compressive strength, stress relaxation damping, deformation, and thermal deformation temperature of the original foam matrix. For instance, Huo et al. [21] carried out injection molding studies to investigate the impact of molding pressure, temperature, and the amount of glass fiber on the properties of PVC materials. Higher pressure resulted in improved molding effectiveness, leading to increased tensile and shear strength. An optimal mold temperature around 100 °C was recommended, as lower temperatures resulted in material looseness and an uneven surface, while excessively high temperatures led to PVC resin degradation and increased ductility. The recommended glass fiber addition was 15 wt.%. Hassan et al. [22] studied the influence of the orientation angle of glass fibers in PVC substrates on material impact resistance. The highest impact compressive strength was observed when the load direction was perpendicular to the glass fibers, whereas the resistance to impact was minimal when the load direction was parallel to the fibers. Fu et al. [23] utilized extrusion and injection molding to investigate the effects of different fiber addition amounts and lengths on product performance. The study indicated that an increase in fiber content significantly reduced the residual fiber length, resulting in a sudden drop in material strength and modulus, making it prone to breakage. Zhao et al. [24] explored the processing of fiber-reinforced PVC composite materials using glass fiber cloth, short glass fibers, and PVC resin as base materials, leading to a substantial improvement in PVC tensile strength, wear resistance, and service life. Kabir et al. [25] proposed the concept of a critical fiber length in their study of fiber-reinforced polymer materials. When fibers are too long their dispersion is poor, leading to aggregation and bending, causing stress concentration, and preventing the achievement of the desired reinforcement effect. Therefore, in practical studies of enhanced modification, it is crucial to optimize fiber content and length parameters.

However, as evident from the aforementioned, the PVC foam modification primarily focused on glass fiber reinforcement. Few research studied involving carbon-fiber-reinforced PVC composite foam (CF/PVC) were reported. Meanwhile, studies have been conducted on the modification of other thermoplastic resins and foams using carbon fiber reinforcement, owing to its significant impact on high specific strength. For instance, Yunus et al. and Li et al. [26,27] investigated the mechanical properties of short-carbon-fiber-reinforced polypropylene composite materials (CF/PP). The optimal tensile strength and bending performance were achieved when the carbon fiber content was 10%, concurrently exhibiting some improvement in wear resistance. Rezaei et al. [28] explored the impact of different carbon fiber lengths on the properties of CF/PP composites. Results indicated that, when the fiber content was constant, composites reinforced with longer fibers exhibited more pronounced improvements in temperature resistance and mechanical properties. Wang et al. [29,30] investigated the influence of CO_2_ saturation pressure and carbon fiber weight percentage on the cell morphology of CF/PP composite foams. At low weight percentages of carbon fiber, a significant portion of the composites remained unfoamed with non-uniform cell size distribution. However, well-defined closed cell structures were observed in the foamed areas. The most uniform cell distribution and morphology were noted with 25 wt.% carbon fiber. Sebaey et al. [31,32] investigated the energy absorption behavior of polyurethane (PU) foam-filled, carbon-fiber-reinforced polymer composite tubes under impact, demonstrating that PU foam is suitable for designing advanced energy absorbers.

This study, targeting the green solution of PVC foam synthetic leather materials for automotive interiors, systematically manipulated the foam formulation, component quantities, and the temperature and time process parameters to investigate their impact on foaming and mechanical properties. The influences of short carbon fibers on the foaming and mechanical properties of PVC foam were further investigated. Ultimately, high-performance CF/PVC composite foam synthetic leather was achieved, characterized by surface density, mechanical properties, and environmental performance that meet the requirements for automotive interior applications, displaying both high performance and low VOC emissions.

## 2. Materials and Methods

### 2.1. Materials

Three types of PVC paste produced via emulsification methods were used for raw matrix resin comparison. The PVC base material of PR-450 (K-value 65) was supplied by Taiwan Plastics Corporation (Ningbo) Limited (Ningbo, China), while 75HV (K-value 73) and 75SK (K-value 72) were provided by Guangzhou Denghua Chemical Co., Ltd. (Guangzhou, China). The plasticizer of diisononyl phthalate (DPHP) was supplied by Xiong County Jinquan Chemical Co., Ltd. (Baoding, China). The foaming agent of azodicarbonamide (ADC) was sourced from Hengshui Jindu Rubber & Chemical Co., Ltd. (Hengshui, China). The zinc oxide foaming promoter, denoted as 311AC, was supplied by Zibo Huaxing Plastic Additives Co., Ltd. (Zibo, China). The acrylic ester processing aid, PA-40, was purchased from Shanghai Kynol Chemical Co., Ltd. (Shanghai, China). The powder calciumzinc stabilizer, known as Stab5, was provided by Guangdong Gaokeda Technology Industry Co., Ltd. (Shantou, China). Short-cut carbon fibers were obtained from Shanghai Lishuo Composite Material Technology Co., Ltd. (Shanghai, China).

### 2.2. Preparation of PVC Foam

A predetermined ratio of PVC base material, plasticizer DPHP (60–80 phr), foaming agent ADC (10–30 phr), processing aid PA-40 (1–4 phr), and heat stabilizer Stab5 (1 phr) were thoroughly mixed and plasticized using a stirring machine (SFJ-400, Huapai Chemical Equipment Co., Ltd., Hefei, China). Subsequently, a vacuum was applied using a vacuum stirrer (ME0089, Getai Machinery Co., Ltd., Dongguan, China) to eliminate air bubbles. The mixed resin paste was poured onto a release paper, and a uniform layer of resin film with a thickness of 0.3 mm was applied using a scraper on the release paper. Foam expansion was carried out on a preheated foaming machine (Jolly, Zhengda Machinery Co., Ltd., Ruian, China) at temperatures ranging from 190 to 215 °C for a foaming duration of 40–90 s. The control of various components of PVC foam and foaming parameters (such as foaming temperature and foaming time) was achieved, and the preparation process of PVC foam was optimized by examining the foam thickness and pore size distribution. The mass ratios of different materials involved in the study were based on the mass of the PVC base material, set as 100 phr.

### 2.3. Preparation of CF/PVC Composite Foam

**Figure 1:**
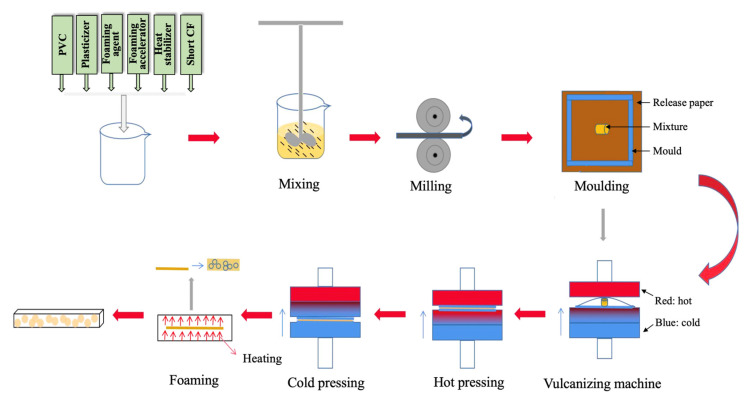
Schematic illustrations of the fabrication process of CF/PVC composite foam using the calendering method.

The short carbon fiber reinforced PVC foam composite materials were prepared using a compression molding method. Optimized material compositions, along with a specific quantity (0–25 phr) of short carbon fibers (0–8 mm), were manually mixed through hand stirring. The pre-mixed materials were then poured into a preheated internal mixer (XH-406BE-50-400, Xihua Testing Instrument Co., Ltd., Dongguan, China) maintained at 150 °C. The front roller speed of the mixer was set at 15 r/min, while the rear paddle speed was set at 10 r/min, and the mixing process continued for 15 min. The resulting block-shaped material was placed into a mold lined with release paper, with a thickness of 0.3 mm. Using a flat vulcanization press (XH-406BE-50-400, Dongguan Xihua Machinery Technology Co., Ltd., Dongguan, China), the material was hot-pressed at 160 °C for 1 min, followed by a cold press for 2 min. Subsequently, the block-shaped material was placed in a preheated coating machine at 195 °C and foamed for 60 s. After the foaming process, the material was removed and cooled to room temperature for 24 h. The simplified preparation process for CF/PVC composite foam is illustrated in Figure 1.

### 2.4. Preparation of PVC Foam Synthetic Leather

**Figure 2:**
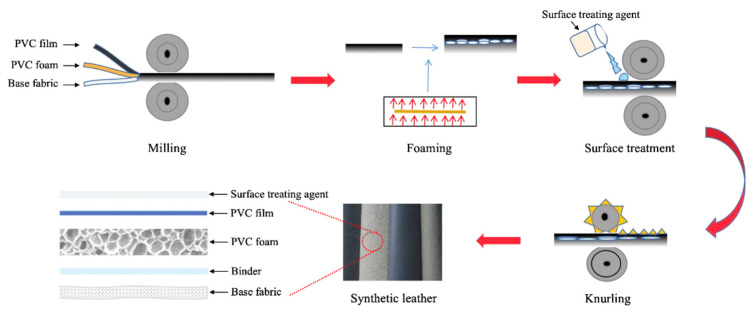
Schematic fabrication process and the layered structure of PVC foam artificial leather.

The preparation process of PVC foam synthetic leather, including CF/PVC composite foam synthetic leather, is depicted in Figure 2.

Blending and pre-plasticization were first performed according to the formulation of PVC foaming material. Subsequently, the mixture was pressed through the hot and cold pressing processes described in Section 2.3 to form a 0.3 mm thick composite material sheet. After a layer of surface PVC film was coated on the sheet in the coating machine, they were heated to 195 °C and maintained for 60 s to shape the surface layer and foam the PVC foaming layer. Then, a surface treating agent of 0.1 mm thick tax was coated on the surface of the foamed layer, and the base fabric was placed flat on the adhesive, put into the coating machine, and heated and dried. Finally, the surface pattern treatment was carried out using water-based polyurethane on a knurling machine after the coating and drying process.

### 2.5. Characterizations and Tests

The apparent density of the foam material was measured using an automatic electronic density meter (BYES-600C, Youzhi Automation Equipment Co., Ltd., Huizhou, China) based on the Archimedes principle buoyancy method according to GMW3182. Foam thickness was measured using a digital thickness gauge (0–10 mm/0.01, Huizhou Xinhui Instrument Equipment Co., Ltd., Huizhou, China), and the foam morphology was observed using a handheld digital microscope (DGX1-AM413, Beijing Haifuda Technology Co., Ltd., Beijing, China). Microscopic morphology was examined using scanning electron microscopy (SEM, Quanta 650, FEI Company, Hillsboro, OR, USA). Image J software (v1.54) was utilized for data collection and analysis of foam pore size distribution, importing microscopic images of the pores into the software for automatic analysis of pore size distribution.

The tensile properties were tested according to the General Motors test standard GMW 3010N [33], using dumbbell-shaped specimens. Tear resistance performance was evaluated according to the ISO 13937-2 standard [34], employing trouser tear specimens for testing. Tensile and tear properties were analyzed using a high–low temperature tensile testing machine (CM2500D, Dawei Xing Technology Co., Ltd., Shenzhen, China) with a test speed set at 100 mm/min, and each sample was tested 3–5 times.

The headspace method, a common test in the automotive industry for benzene-type volatile organic compounds, was used to evaluate the VOC emission from PVC foam synthetic leather according to GMW3205. Specifically, 2 g of PVC foam synthetic leather was placed in a 20 mL headspace bottle and kept at 120 °C for 60 min. A certain amount of gas was then withdrawn from the headspace bottle using a syringe, and the withdrawn gas was analyzed using a gas chromatograph (7890A GC, Agilent, Santa Clara, CA, USA) to assess VOC emissions.

## 3. Results and Discussion

### 3.1. Properties of PVC Foam

#### 3.1.1. The Influence of PVC Raw Materials on Foaming

**Figure 3:**
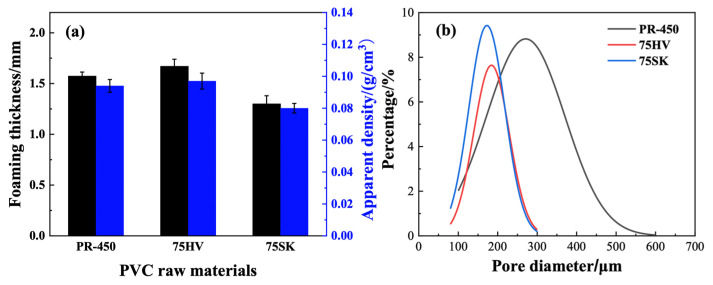
Foaming properties of different PVCs: (**a**) Foaming thickness and apparent density; (**b**) Pore size distribution.

As depicted in Figure 3a, the sequence of foaming thickness and apparent density for three different PVC types is 75HV > PR-450 > 75SK.

**Figure 4:**
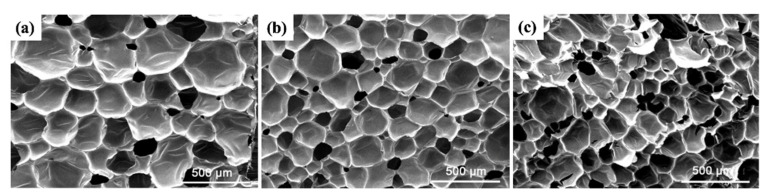
SEM images of different PVC foams: (**a**) PR-450; (**b**) 75HV; (**c**) 75SK.

Generally, larger foaming thickness should result in smaller apparent density of the foam. However, in this study, as the foaming thickness of PVC increases its apparent density also increases. This is attributed to the positive correlation between the density of PVC, serving as the base material for foaming, and the density of the foamed material. The density of PVC powders follows the order of 75HV > PR-450 > 75SK, consequently influencing the apparent density sequence of the foamed materials, which is also 75HV > PR-450 > 75SK. The SEM images (Figure 4) of the pore structure after foaming for the three PVC types were imported into the Image J software v1.54, and the results of the pore size distribution are shown in Figure 3b.

The study indicates that the pore size distribution for PR-450 foaming is between 100 and 600 μm, mainly concentrated in the range of 200–400 μm. For 75HV and 75SK foaming, the pore size distribution is within 100–300 μm, predominantly centered around 100–250 μm. Due to the higher viscosity of 75HV resin paste compared to 75SK resin paste, it inhibits pore overflow. Consequently, 75HV foaming material has more pores internally than 75SK, contributing to the larger foaming thickness of 75HV. Considering both foaming thickness and pore size distribution, 75HV is selected as the preferred PVC raw material for subsequent research.

#### 3.1.2. The Influence of Functional Additives on PVC Foaming

**Figure 5:**
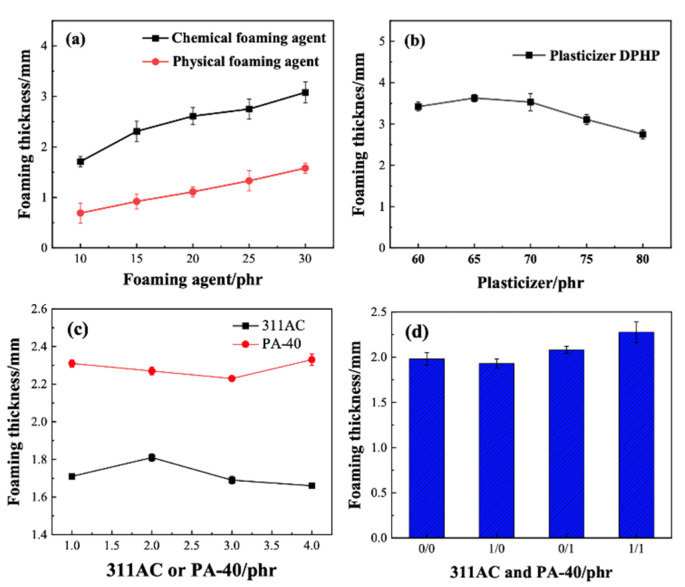
Influence of the material and content of the foaming agent (**a**), plasticizer (**b**), accelerator 311AC and processing agent PA-40 (**c**), and 311AC/PA-40 content on the foaming thickness of PVC foams (**d**).

The PVC foaming process involves three main steps: bubble nucleus formation, growth, and stabilization. During foaming, the system undergoes rapid depressurization or heating, causing an increase in viscosity and expansion of the melt, leading to the generation of numerous gas nuclei under these unstable conditions. The type and amount of foaming agent are crucial factors influencing the foaming performance of PVC foam materials. Therefore, selecting appropriate foaming agents and their concentrations can produce materials with increased foaming thickness and reduced weight. Investigating the effect of foaming agent concentration on PVC foaming, different amounts of foaming agents were introduced. The results indicate that the chemical foaming agent ADC performs better than physical foaming agents, as shown in Figure 5a.

An increase in foaming agent concentration leads to an increase in the number of pores, accompanied by the phenomenon of pore fusion and enlargement. In other words, the quantity of large pores and foaming thickness increases. Too little foaming agent results in insufficient foaming thickness, while excessive amounts lead to undesirable coalescence and stringing between pores, causing material damage.

To investigate the influence of plasticizer dosage on PVC foaming characteristics, different amounts of plasticizer were added to PVC foam materials. As shown in Figure 5b, with the increase in plasticizer content, the foaming thickness initially increases and then decreases. The presence of the plasticizer reduces the viscosity of the PVC melt, promoting the growth of pores and making them expand more easily after pressure removal, thereby increasing the foaming thickness and volume [5]. However, when the dosage of DPHP is relatively high there is a noticeable decrease in foaming thickness. Excessive addition of plasticizer can result in excessively low viscosity of the PVC melt, reducing the hindrance to pore growth. This may lead to phenomena such as pore adhesion, coalescence, and increased collapse between pores [35]. Through experiments, it was determined that adding 65 phr of DPHP plasticizer could produce PVC foam materials with better foaming effects, achieving a foaming thickness of up to 3.63 mm.

ADC is a commonly used foaming agent, but its dissolution temperature is relatively high. For low decomposition temperature materials like PVC, this can lead to issues such as temperature mismatch and uneven pore distribution. To address these problems, foaming additives can be added to optimize the performance of ADC. As shown in Figure 5c, the research results indicate that adding an appropriate amount of foaming additive (331AC) can enhance the foaming performance of PVC. However, with the increase in processing aid (PA-40) content, the foaming thickness of PVC initially decreases and then increases. This is because the increased content of processing aid leads to frictional heat generation, resulting in higher temperature and shorter resin melting time, which reduces foaming thickness. However, when the addition amount is 4 phr, the increased temperature and rapid resin melting lead to larger pores and increased foaming thickness. Under different 311AC/PA-40 ratios (Figure 5d), when 311AC/PA-40 is 1/1 PVC exhibits the best foaming performance, achieving a foaming thickness of 2.31 mm, better than the cases without additives or with only one type of additive. Through comprehensive analysis, the optimal solution is to add 1 phr of foaming additive and 1 phr of processing aid.

Based on the above exploration, the preferred formulation for PVC foaming materials is presented in Table 1.

#### 3.1.3. The Influence of Foaming Process on PVC Foaming

Foaming temperature and time are the two crucial parameters affecting PVC foam pore parameters. This is attributed to the fact that the process of foaming agent decomposition does not occur instantaneously and requires heating and a certain amount of time.

**Figure 6:**
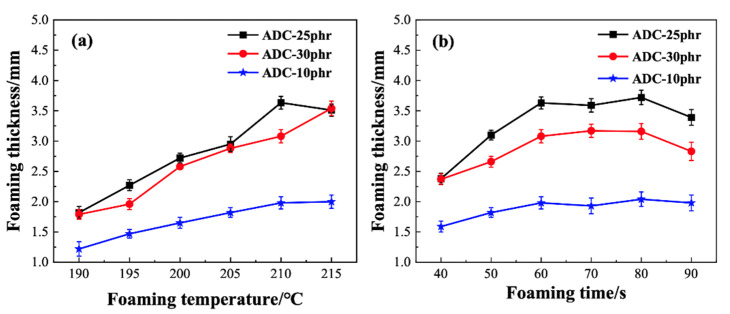
Influence of foaming temperature (**a**) and foaming time on the foaming thickness (**b**) of PVC foams.

In order to investigate the effect of PVC foaming temperature on the foaming thickness, experiments were conducted using PVC resin paste with different foaming temperatures but the same foaming time. In the actual production of products, a low heating temperature may result in the foaming ratio of the product not meeting the requirements, with fewer formed pores, and the product’s elasticity being relatively poor. Conversely, a high heating temperature may cause the premature decomposition of the foaming agent, leading to the foaming thickness of the foamed material not reaching the expected level [36]. As shown in Figure 6a, foaming was carried out on the PVC resin paste at temperatures ranging from 190 °C to 215 °C, and the foaming thickness was tested.

The results indicate that with the increase in foaming temperature the foaming thickness also increases. When the foaming agent ADC content is 25 phr and the foaming temperature is 210 °C the foaming thickness reaches its maximum value. This is because, with the increase in temperature, the decomposition rate of the foaming agent accelerates, leading to a faster generation of gas, thereby increasing the foaming thickness.

Utilizing a constant foaming temperature of 210 °C, we investigated the influence of foaming time on the foaming performance of PVC. As shown in Figure 6b, with an increase in foaming time from 40 s to 90 s, the foaming thickness exhibited a gradually increasing trend. Specifically, from 40 s to 60 s the foaming thickness of PVC increased rapidly, while after 60 s the increase became less pronounced; in some cases, a decrease in foaming thickness was observed. This phenomenon is attributed to the gradual decomposition of the ADC foaming agent at 210 °C, releasing gas that forms within the matrix, achieving the foaming effect. As the foaming heating time increases, the decomposition rate of the foaming agent accelerates, and the speed of gas generation also increases. Due to the sharp increase in the number of bubbles, the foaming expansion rate rapidly increases, leading to an increase in foaming thickness. However, when the decomposition of the generated gas ceases upon further heating, the already-formed PVC pores may stick together due to the influence of heat, resulting in phenomena such as coalescence and collapse of the foam structure, leading to a reduction in foaming thickness. Therefore, we recommend selecting the optimal foaming temperature and time as 210 °C and 60 s, respectively.

### 3.2. Properties of CF/PVC Composite Foam

#### 3.2.1. The Influence of Carbon Fibers on PVC Foaming

**Figure 7:**
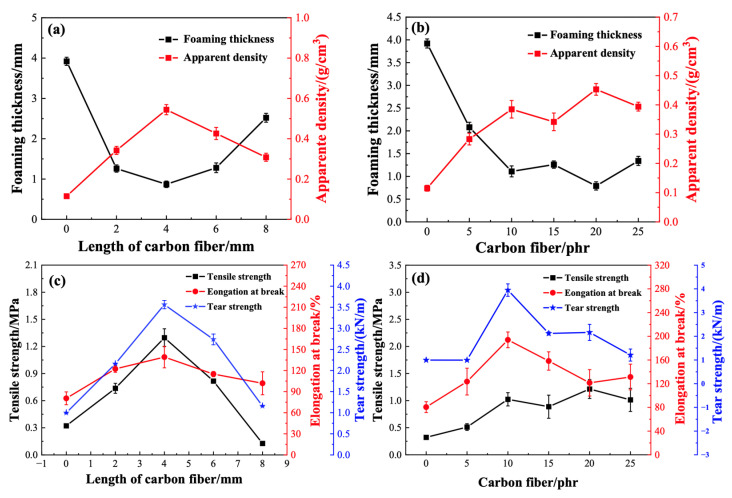
Influence of length and content of short carbon fibers on the foaming thickness and apparent density (**a**,**b**) and mechanical properties (**c**,**d**) of PVC foams.

We employed four different lengths of carbon fibers (2 mm, 4 mm, 6 mm, and 8 mm) to reinforce PVC foamed materials and examined their foaming quality and apparent density. As depicted in Figure 7a with 8 phr carbon fibers, the increase in carbon fiber length has an impact on the foaming quality of PVC foamed materials.

Specifically, with the elongation of carbon fiber length, the foaming thickness of PVC foamed materials initially decreases and then increases, while the density exhibits an initial increase followed by a decrease. This phenomenon arises due to the introduction of short carbon fibers, which facilitates the formation of numerous gas nuclei alongside the carbon fibers. During the growth of gas nuclei, hindered by the presence of carbon fibers, the bubbles are less likely to expand, ultimately resulting in smaller pore diameters and reduced material foaming thickness. When adding carbon fibers with lengths greater than 4 mm, the foaming extent of PVC foamed materials increases with the elongation of the carbon fiber length. This is because longer added carbon fiber lengths result in the formation of more gas nuclei, which reach saturation earlier and are prone to overflow and expansion. Figure 7b illustrates the impact of using different short carbon fibers on the foaming thickness and density of PVC foamed materials. The foaming thickness exhibits a fluctuating decreasing trend with the increase in short carbon fiber content, while the apparent density demonstrates an opposite trend with an increase in fiber content.

#### 3.2.2. The Influence of Carbon Fibers on the Mechanical Properties of PVC Foam

To investigate the impact of adding short-cut fibers on the mechanical properties of PVC foam materials, various lengths and quantities of short-cut carbon fibers were incorporated into PVC foaming materials, and their changes in mechanical properties were recorded. As depicted in Figure 7c, with the increase in carbon fiber length the strength of PVC foaming materials initially increases and then decreases. Specifically, when the added carbon fiber length is 4 mm, the tensile strength, fracture elongation, and tear strength are 1.30 MPa, 139%, and 3.56 kN/m, respectively. For carbon fiber lengths less than 4 mm, the increase in fiber length results in a greater enhancement of the mechanical strength of PVC foam. This is because, with a moderate increase in carbon fiber length, the contact area between short-cut carbon fibers and PVC increases, reinforcing the interface interaction and requiring greater tension upon fracture, thereby leading to increased mechanical strength. However, when carbon fiber lengths exceed 4 mm, a significant decrease in material strength is observed. This is attributed to the entanglement and agglomeration of excessively long carbon fibers, causing uneven distribution within the resin matrix. Consequently, areas with entangled carbon fibers are more prone to fracture under external forces, resulting in a decline in material strength.

Continuing the exploration using short-cut carbon fibers with a length of 4 mm, we examined the influence of fiber content on the properties of PVC foam materials. Figure 7d illustrates the relationship between tensile strength, tear strength, fracture elongation, and the added amount of short-cut carbon fibers. Tensile strength shows a gradual increase with the rise in short-cut carbon fiber content. However, tear strength and fracture elongation exhibit an initial increase followed by a decrease with the increase in short-cut carbon fiber content, reaching maximum values of 3.95 kN/m and 194%, respectively, at a fiber addition of 10 phr.

**Figure 8:**
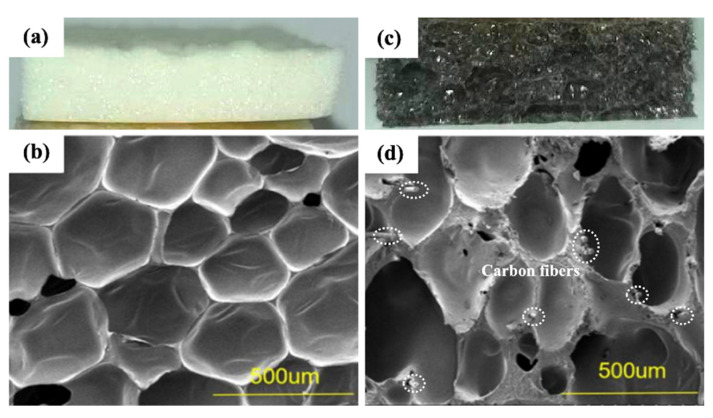
Digital and SEM images: (**a**,**b**) PVC foam; (**c**,**d**) CF/PVC composite foam.

To explore the reasons behind the enhancement in mechanical properties with fiber addition, we conducted SEM observations of the PVC foam after mechanical property testing, as shown in Figure 8.

After adding short-cut carbon fibers, the morphology of PVC foam materials remained similar to that before addition, characterized by nearly circular closed cells and a small number of open cells. Short-cut carbon fibers were mainly distributed on the cell wall, reinforcing the cell wall strength. Under the internal stress of pure foam, once a foam column ruptures, adjacent foam walls also rupture, and cracks rapidly propagate to nearby foam. In fiber reinforced foam, when cracks widen to the fibers, the presence of fibers causes crack termination and deviation, reducing the internal stress of cracks and enhancing the overall mechanical properties of PVC foam materials.

### 3.3. CF/PVC Composite Foam Synthetic Leather

#### 3.3.1. Areal Density and Mechanical Properties

Table 2 provides a comparison of the areal density, tensile properties, and tear resistance of synthetic leathers produced from different PVC foam materials with automotive standard values.

- Areal density: The areal density of automotive PVC foam synthetic leather should be below 800 ± 80 g/m^2^ [37]. In this study, the average areal densities of the PVC foam synthetic leather and CF/PVC composite foam synthetic leather prepared were 765 g/m^2^ and 773 g/m^2^, respectively, meeting the requirements.
- Tensile properties: The tensile strength of synthetic leather made from PVC foam material is 32.6 MPa. In contrast, the tensile strength of synthetic leather prepared from CF/PVC composite foam increased to 48.9 MPa, a 50% improvement. This is attributed to the increased tensile strength of the foamed layer, and the addition of short fibers results in a rougher sample surface, enhancing the bonding between the PVC foam layer and other layers of PVC synthetic leather, thereby improving the overall tensile strength. However, the break elongation of synthetic leather prepared using CF/PVC foam material is reduced. Specifically, the elongation at break for synthetic leather prepared from PVC foam material is 214%, while for CF/PVC composite foam it decreases to 185%. The addition of carbon fibers reduces the mobility of PVC molecular chains during deformation, resulting in decreased plasticity, ultimately leading to a reduction in the elongation at break of PVC synthetic leather and a slight decrease in flexibility. Nevertheless, automotive-grade PVC foam synthetic leather requires a tensile strength greater than 35 MPa and a break elongation of 180–215% [33]. The tensile strength and break elongation of CF/PVC composite synthetic leather in this study still comply with the specified criteria.
- Tear resistance: The tear strength of synthetic leather prepared from neat PVC foam material is 11.4 kN/m, whereas synthetic leather prepared from CF/PVC composite foam material shows an increased tear strength of 14.7 kN/m. This improvement is attributed to the addition of fibers, which hinder the expansion of cracks, requiring greater tearing force to rupture PVC foam synthetic leather and, thus, enhancing tear resistance. Automotive-grade PVC foam synthetic leather requires tear strength greater than 10 kN/m [34], and the tear strength of CF/PVC foam synthetic leather in this study also meets the requirements.

#### 3.3.2. VOC Emissions Evaluation

We carried out tests for “three aldehydes and five benzenes” and the overall VOC volatilization situation. Table 3 presents the VOC emission characteristics of two types of PVC foam synthetic leather.

The VOC tests for synthetic leather produced using PVC foam and CF/PVC composite foam both met the requirements [38]. Although the addition of carbon fibers resulted in a slight increase in the release of aldehydes and benzene VOCs in CF/PVC composite foam synthetic leather, the total VOC volatilization decreased from 5.505 mg/m^3^ for pure PVC foam to 3.975 mg/m^3^. This may be attributed to the fact that adding fibers helps promote bubble nucleation, increasing micropores in the foam, which is conducive to the volatilization and emission of VOCs. However, at the same time, the inclusion of carbon fibers to some extent restricts the movement of PVC molecular chains, enhances the thermal stability of the foaming material, and delays aging and thermal decomposition under high-temperature conditions, thereby reducing the total VOC emissions. Similar results were also observed in hemp-fiber-reinforced polypropylene composites, where the VOC emissions reduction was attributed to the thermal stability enhancement through fiber reinforcement, as evidenced by the differential thermal analysis (DTA) and thermogravimetric analysis (TGA) [39]. Gu et al. [40] clearly showed that PU composite foam had improved cell size and higher glass transition temperature (T_g_) due to the presence of wood fiber characterized by thermal gravimetric analysis (TGA) and differential scanning calorimetry (DSC) results. Furthermore, the carbon fiber inducing polymer thermal stability enhancement can be widely found in traditional fiber-reinforced thermoplastic composites such as CF/PP, CF/Nylon, etc. [19,41]. Therefore, in this paper, the addition of CF not only enhances the mechanical properties of PVC foam as expected, which can reduce VOC emissions due to the PVC usage reduction attributed to the high specific strength of CF/PVC itself, but also directly enhances the thermal stability of PVC foam, which further lowers the level of VOC emissions during its applications. In conclusion, the addition of carbon fibers plays a dual role in positively reducing VOC emissions from PVC foam when applied in synthetic leather.

## 4. Conclusions

This study aimed to enhance the performance of PVC foam materials and reduce VOC emissions. The optimization of the PVC foam material formulation and foaming process, coupled with the incorporation of short carbon fiber reinforcement technology, was undertaken to produce PVC foam and CF/PVC composite foam synthetic leather with comprehensive and application-compliant properties. The key conclusions are as follows:

1. The types and ratios of PVC raw materials, plasticizers, foaming agents, and processing aids significantly impact the foaming performance of PVC foam materials. The optimal foaming formulation for PVC was determined as follows: using 75HV as the base material, adding 65 phr plasticizer, 25 phr foaming agent, 1 phr foaming assistant, 1 phr processing aid, and 1 phr thermal stabilizer.
2. The tensile strength and tear strength of CF/PVC composite foam synthetic leather were improved compared to PVC foam synthetic leather, increasing from 32.6 MPa to 48.9 MPa and from 11.4 kN/m to 14.7 kN/m, respectively. Although the elongation at break decreased from 214% to 185%, it still met the requirements for automotive interior applications. High performance implies reduced PVC usage and lower VOC emissions.
3. Due to the hindering effect of carbon fiber on PVC molecular chain movement, the aging of PVC foam was inhibited, resulting in a reduction in total VOC emissions from 5.505 mg/m^3^ for PVC foam synthetic leather to 3.975 mg/m^3^ for CF/PVC composite foam synthetic leather.
4. The CF/PVC composite foam synthetic leather production process presented in this study demonstrates excellent scalability. Future endeavors could focus on optimizing cost implications to enhance its market competitiveness, for instance, by incorporating recycled carbon fibers.

## Figures and Tables

**Table 1:** Optimized compounds of foaming PVC.

Material	Role	Weight Ratio (phr)
PVC	Resin	100
DPHP	Plasticizer	65
ADC	Foaming agent	25
311AC	Foaming accelerator	1
PA-40	Processing agent	1
Stab5	Heat stabilizer	1

**Table 2:** Areal density and tensile property of artificial leather fabricated using different PVC foams.

	PVC Foam	CF/PVC Foam	Requirements
Areal density (g/m^2^)	765	773	800 ± 80 [37]
Tensile strength (MPa)	32.6	48.9	>35 [33]
Elongation at break (%)	214	185	180–215 [33]
Tear strength (kN/m)	11.4	14.7	>10 [34]

**Table 3:** VOC emissions of PVC and CF/PVC foam synthetic leather (Units: mg/m^3^).

PVC Foam Materials	Methanal	Acrolein	Acetaldehyde	Benzene	Methylbenzene	Styrene	Xylene	Ethylbenzene	Total VOC ^1^
PVC foam	0.005	0.000	0.022	0.002	0.019	0.000	0.000	0.000	5.505
CF/PVC foam	0.009	0.000	0.030	0.005	0.025	0.000	0.029	0.000	3.975
Requirements [38]	≤0.10	≤0.05	≤0.05	≤0.11	≤1.10	≤0.26	≤1.50	≤1.50	

^1^ The “Total VOC” not only includes aldehydes and benzene but also encompasses plasticizers, substances generated from PVC aging itself, various stabilizers, materials produced by the thermal decomposition of ADC foaming agent, and the cumulative sum of some chlorine-containing small molecules.

## Data Availability

Data are contained within the article.
